# Case of Hemorrhagic Diathesis Following Supprsssion of the Catamenia, and Attended by Vicarious Discharge from the Gums—Terminating Fatally from Hemorrhage Following Scarification

**Published:** 1850-07

**Authors:** 


*Case of Hemorrhagic Diathesis following Supprsssion of the Catamenia, and attended by Vicarious discharge from the Gums—terminating fatally from hemorrhage following scarification.* By I. B. Dunlap, M. D., of Norristown, Pa.

About the 1st of February last, I was called upon, during the absence of her physician, to visit a young lady about twenty-two years of age, who was discharging blood profusely from the mouth, and who, judging from her appearance, was laboring under considerable hepatic derangement. The attack of hemorrhage had commenced four or five days previous, and had continued without intermission up to the moment that I first saw her. On making inquiry into her previous condition, I was informed that she had not enjoyed good health for the past year, but had labored under more or less acute continuous pain in the right hypochondrium, together with considerable irregularity in her menstrual discharge. For the relief of these difficulties, she had been under medical treatment, but derived no permanent benefit from the advice and prescriptions of her attendants. She remained in this condition for about six months, when she became completely jaundiced, and her catamenia were entirely suppressed. Six weeks from the period at which she last observed any appearance of the monthly discharge, she was attacked with severe pain across the loins, nausea, and a profuse discharge of blood from the mouth; her physician was sent for, who made use of the ordinary remedies for arresting hemorrhage, but without effecting any diminution in the discharge, which continued without intermission for five days, and then suddenly ceased. After this, she enjoyed tolerably good health, suffering no uneasiness with the exception of the pain in her side, until the expiration of a month, when she was again attacked with hemorrhage, and which, under medical treatment, ran its course, as before, uncontrolled. For the last four months she has had these periodical attacks of bleeding, enjoying comparatively good health during the intervals.

Having taken charge of the case by the request of the family, I was determined to watch it closely, and ascertain, if possible, from whence the hemorrhage proceeded. I therefore requested the family to apprise me the moment that the premonitory symptoms of a return of bleeding should present themselves. Accordingly, in the course of a few days I was sent for, and upon opening the patient's mouth, saw distinctly the blood oozing from the gums in front of and behind the teeth, and as far back as the first molars, beyond which I am certain that no blood proceeded. By wiping off the gums the blood in an instant would again swelter out, covering them completely, and by making pressure upon them, blood could be forced out in increased quantities. I may here remark, that the blood was neither thrown out by hawking, coughing, or vomiting, but discharged by incessant spitting. The bleeding continued to increase, as it had done on other occasions, in spite of all remedial applications, for five or six days, when it spontaneously subsided. During each of these paroxysms of hemorrhage, there was discharged, as near as we were able to ascertain, about six quarts of blood. I now endeavored to restore her catamenia, and correct, if possible, the derangement of the liver. To meet these ends the patient was put upon the use of the sulph. ferri exsiccat. with aloes, alternated with blue mass combined with the extracts of taraxacum and gentian; at the same time, counter-irritants were freely applied over the region of the liver, and the nitro-muriaric acid foot-bath made use of every night at bedtime. This course was pursued for four weeks, when a return of bleeding from the gums again took place. This continued some two or three days longer than usual, and the patient was evidently more prostrated from this loss of blood than from any of the previous ones. Port wine with tinct. rhatany, was freely given, and the bleeding appeared to diminish, when, unfortunately for herself, during my absence, on account of the severe pain in her side, and by the advice of a meddlesome friend, she sent for a cupper, who applied some half a dozen cups on the right hypochondrium, the consequence of which was that the bleeding from the gums entirely ceased, but it was found impossible, by the use of any means, to arrest the hemorrhage from the scarifications. She soon became pulseless, and in less than six hours died. To satisfy myself in regard to the source of the bleeding, I called in my worthy friend Dr. H. D. W. Pauling, who, after examining the case, fully concurred with me in opinion.

*Autopsy.*—Shortly after death, an examination of the body was made, and the thoracic and abdominal viscera carefully examined. The contents of the thorax were in a perfectly normal condition, and the viscera of the abdomen, with the exception of the liver and ovaries, were in a similar state. The latter organs were somewhat enlarged. In the gall bladder were found seven calculi, each about the size of a hazel nut, of an octangular form, with their faces ground perfectly smooth by rubbing against each other.

I regard this as an interesting case in several particulars. Here was evidently a vicarious hemorrhage, a substitute for the catamenia, and this proceeded altogether from apparently healthy gums, and from but a very minute surface when compared with the large amount of blood thrown out. Again, there was certainly a hemorrhagic condition of the system, which was not at all amenable to remedial agents, as was shown by all the means made use of to arrest the bleeding from the gums, or scarification, all of which proved abortive. But does it not appear remarkable, that the moment the cups began to fill, the bleeding from the gums should cease entirely? We are aware of the fact that blood-letting frequently acts as a derivative in hemorrhage, but never before saw a case where it produced such an instantaneous stoppage to the original bleeding, nor of its occasioning fatal results in consequence of the inability to arrest it.—*N.* Y. *Jour. of Med.*

